# Sarcoidosis-Like Reaction in a Patient With Psoriatic Arthritis Treated With Adalimumab

**DOI:** 10.7759/cureus.76563

**Published:** 2024-12-29

**Authors:** João Oliveira, Martina Arandjelovic, Inês Pintor, Margarida Resendes, Flávio Pereira

**Affiliations:** 1 Internal Medicine, Hospital Infante D. Pedro, Aveiro, PRT; 2 Infectious Diseases, Hospital Infante D. Pedro, Aveiro, PRT

**Keywords:** adalimumab (humira), anti-tnf agents, drug-induced sarcoidosis-like reaction, psoriatic arthritis (psa), tumor necrosis factor

## Abstract

A drug-induced sarcoidosis-like reaction (DISR) is a systemic granulomatous reaction indistinguishable from sarcoidosis and is associated with the administration of a medication. It typically exhibits a temporal relationship with the initiation of the drug (an average interval of 22 months) and tends to improve upon its discontinuation. Tumor necrosis factor (TNF) antagonists, including adalimumab, have been associated with the development of DISR. The case presented here describes a 33-year-old female patient with psoriatic arthritis treated with adalimumab, whose clinical presentation, imaging findings, and histology were consistent with DISR.

## Introduction

Tumor necrosis factor (TNF) is a pleiotropic cytokine with both homeostatic and pathogenic activities. Its homeostatic functions include defense against pathogens, involvement in organogenesis, tissue regeneration, immunoregulation, and inhibition of tumorigenesis. However, TNF also has pathogenic roles, notably its participation in inflammation, autoimmunity (through the inhibition of regulatory T lymphocytes), tissue degeneration, hypernociception, tumorigenesis, and atherogenesis [1,2].

Approved anti-TNF agents represent a scientific breakthrough that has significantly improved the quality of life of millions of people worldwide with immune-mediated diseases. Nevertheless, these drugs present certain challenges, including numerous adverse events such as reactivation of latent tuberculosis, development of opportunistic infections, paradoxical induction of autoantibodies, sarcoidosis-like symptoms, and an increase in cancer risk [1].

Adalimumab is a recombinant human anti-TNF monoclonal antibody used in the treatment of various immune-mediated diseases such as psoriatic arthritis, rheumatoid arthritis, and Crohn’s disease [3]. Since TNF plays a crucial role in the formation and maintenance of sarcoid granulomas, adalimumab and other anti-TNF agents are used in the treatment of sarcoidosis [2,4]. Curiously, however, these TNF antagonists can also induce a paradoxical response known as a sarcoidosis-like reaction [4]. A drug-induced sarcoidosis-like reaction (DISR) is a systemic granulomatous tissue response that is clinically and histologically indistinguishable from sarcoidosis and presents a strict temporal relationship with the start of the new medication (an average interval of 22 months). In general, the reaction improves or resolves upon discontinuation of the drug in question [1,4,5]. The exact mechanism underlying the anti-TNF-induced DISR remains unclear. It is believed that TNF plays a role in activating and suppressing various cellular signaling pathways depending on the specific inflammatory milieu [5]. Considering the importance of DISR as a secondary effect of the administration of anti-TNF agents, we present the case of a 33-year-old woman with psoriatic arthritis treated with adalimumab, consistent with DISR.

## Case presentation

**Figure 1:**
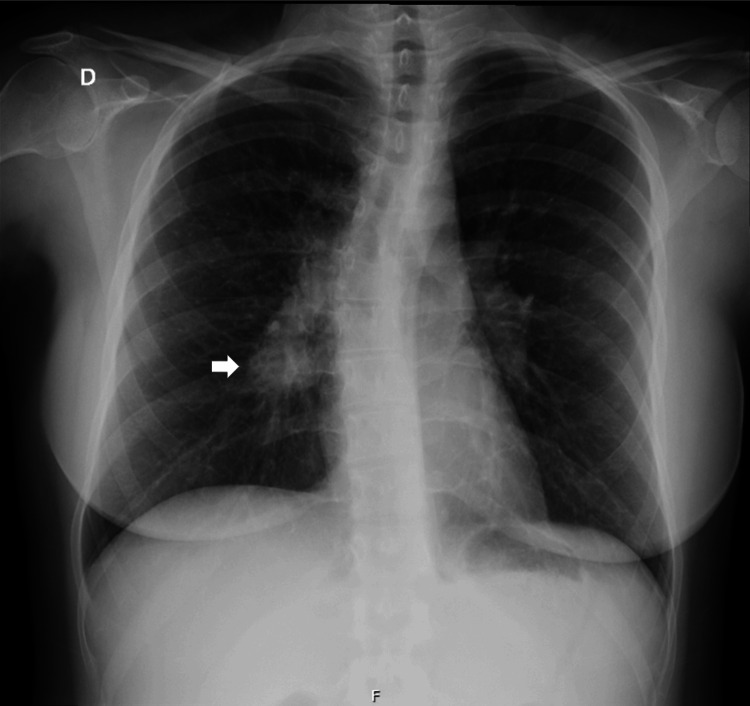
Posteroanterior chest X-ray. Performed on May 17, 2024, in the Emergency Department depicting right juxtahilar nodular hypotransparency (arrow).

The authors present the case of a 33-year-old female patient with a medical history of psoriatic arthritis, treated with weekly methotrexate 15 mg and fortnightly adalimumab 40 mg since January 2023. In May 2024, the patient presented to the emergency department with a three-week irritating cough that had worsened over the preceding three days, accompanied by a low-grade fever (37.7°C axillary), asthenia, and thoracic back pain with pleuritic characteristics. On examination, she was hemodynamically stable, with a tympanic temperature of 37.5°C and peripheral oxygen saturation of 98% on room air. Pulmonary auscultation was unremarkable. Blood tests revealed 11.3 x 10⁹/L leukocytes (4.1-11.1 x 10⁹/L), 7.88 x 10⁹/L neutrophils (2.0-7.5 x 10⁹/L), and 11.7 mg/dL C-reactive protein levels (0-0.5 mg/dL). Despite extensive investigation, no causative agent was identified. Serological testing for the human immunodeficiency virus (HIV) was negative. A chest X-ray showed a right juxtahilar nodular hypotransparency (Figure 1). The diagnosis of community-acquired pneumonia caused by an unidentified microorganism was assumed in a patient without respiratory failure and undergoing immunosuppressive treatment for a rheumatological disease. She was admitted to the hospital-at-home program and completed a course of antibiotic therapy with amoxicillin-clavulanic acid and azithromycin. Methotrexate and adalimumab were suspended.

**Figure 2:**
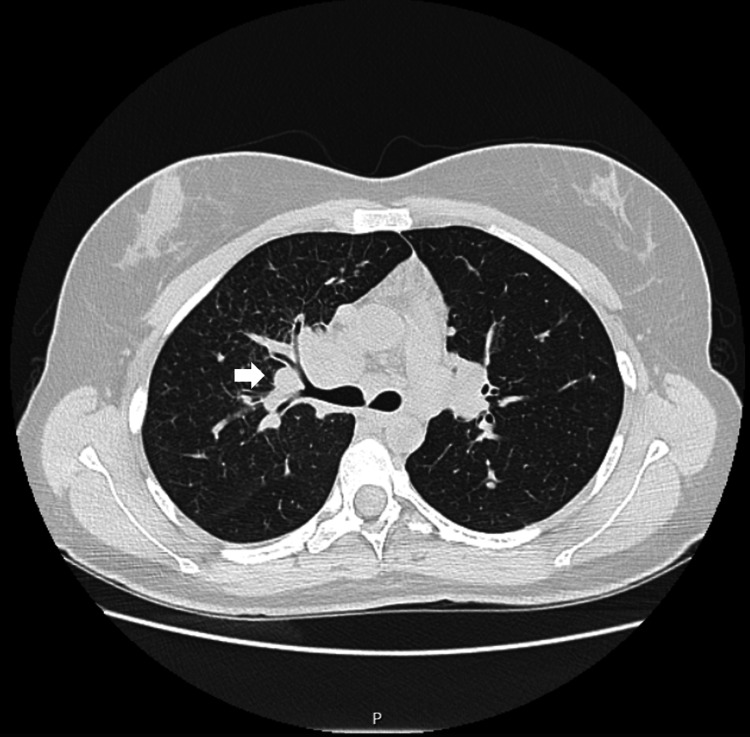
First chest computed tomography scan showing the hilar lymphadenopathy clusters marked with an arrow (May 28, 2024).

The patient showed favorable clinical and analytical evolution and was discharged with follow-up in outpatient care. A chest computed tomography (CT) scan was also requested to further investigate the radiographic findings. At her follow-up appointment one month after discharge, she reported no respiratory symptoms but exhibited a slight worsening of nail psoriasis. The chest CT scan (Figure 2) revealed lymphadenopathy clusters located in the pulmonary hila, with the right side being more prominent, measuring 51 x 32 mm in the axial plane. Additional findings included areas of increased lung parenchyma density, interlobular septa thickening in the right upper lobe, and scattered bilateral micronodules with an apical-caudal distribution. Based on these findings, the differential diagnoses included lymphoproliferative disease, pulmonary tuberculosis, and an adalimumab-induced sarcoidosis-like reaction. Further investigation showed an angiotensin-converting enzyme (ACE) level of 39.8 U/L (20.0-70.0 U/L), a negative interferon-gamma release assay (IGRA) result, and a negative antineutrophil cytoplasmic antibodies (ANCA) test.

**Figure 3:**
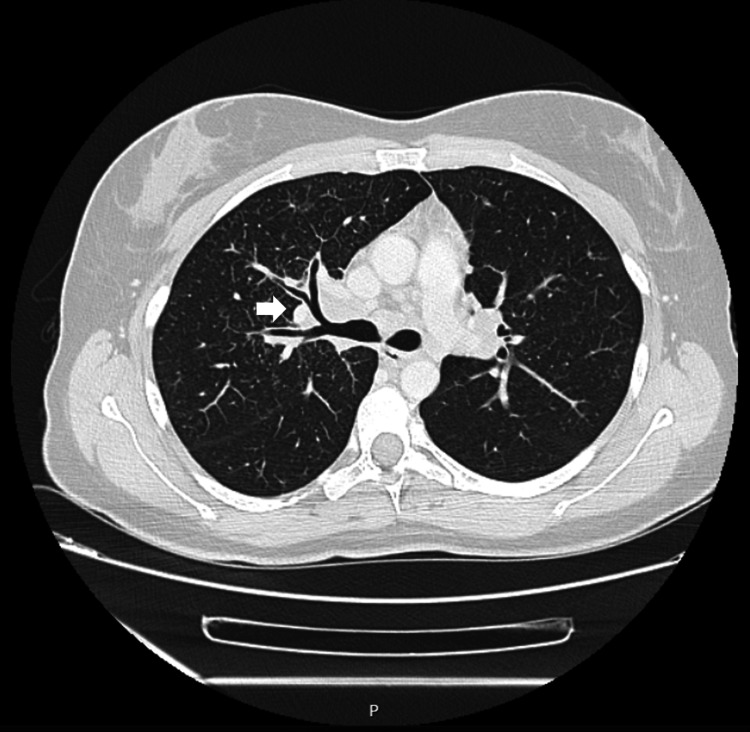
Second chest computed tomography scan showing the progression of the hilar lymphadenopathy clusters marked with an arrow (July 18, 2024).

A follow-up chest CT scan (Figure 3), performed two months after the initial CT imaging, showed a reduction in the right hilar lymphadenopathy conglomerate to 37 x 18 mm in the axial plane. However, the interlobular septa thickening and scattered bilateral micronodules persisted.

**Figure 4:**
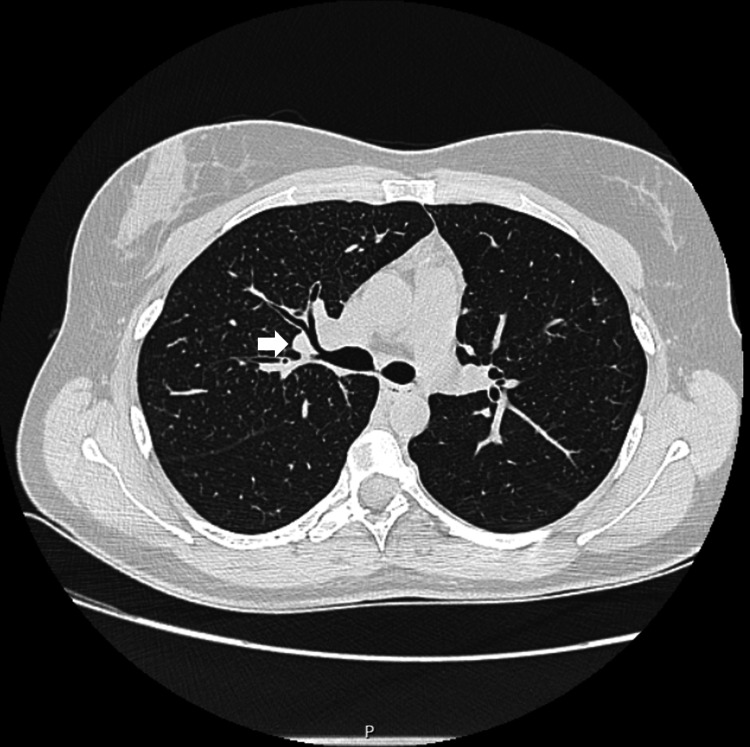
Third chest computed tomography scan showing the progression of the hilar lymphadenopathy clusters marked with an arrow (October 17, 2024).

The patient underwent an endobronchial ultrasound (EBUS), during which a transbronchial biopsy of the lymphadenopathy conglomerate was performed along with bronchoalveolar lavage (lymphocytes 44%; CD4/CD8 ratio 5.9), and bronchial aspirate for mycobacterial culture (negative). Cell analysis by flow cytometry and immunophenotyping revealed no significant abnormalities. Histological examination revealed a lymph node with non-caseating epithelioid granulomas and no evidence of metastatic neoplasia. The diagnosis of an adalimumab-induced sarcoidosis-like reaction was established. Five months after the discontinuation of adalimumab, a repeat chest CT scan (Figure 4) showed no mediastinal lymphadenopathy and only diffuse micronodularity in the upper lobes, consistent with sarcoidosis. The patient is currently asymptomatic and is receiving treatment with secukinumab 300 mg weekly.

## Discussion

In the presented case, the clinical findings (cough, fever, and pleuritic pain) combined with the analytical and imaging results in an immunocompromised patient initially led to a diagnosis of community-acquired pneumonia. Indeed, pneumonia and sarcoidosis have many overlapping signs and symptoms [6]. The first chest CT scan (Figure 2) raised the possibility of sarcoidosis or a sarcoidosis-like reaction. The imaging findings observed in this case align with those described in the literature for sarcoidosis, including bilateral hilar lymphadenopathy, scattered micronodules predominantly in the upper and middle thirds of both lungs, irregular septal thickening, and areas of increased lung parenchyma density [7]. In this case, the patient’s serum ACE levels were 39.8 U/L, which is within normal limits. The literature indicates that approximately 60% of patients present with elevated ACE levels at the time of diagnosis [8]. Given that the patient was immunocompromised (on methotrexate and adalimumab therapy), an IGRA test was performed to rule out *Mycobacterium tuberculosis* infection, which returned negative. Testing for ANCA was also negative, excluding the possibility of vasculitis. Histological examination ruled out neoplasia and identified non-caseating epithelioid granulomas-a key diagnostic feature of sarcoidosis [7].

Two aspects supported the diagnosis of a sarcoidosis-like reaction, whose immunopathogenesis remains uncertain. On the one hand, there was a temporal association between the initiation of the anti-TNF agent and the onset of symptoms (approximately 15 months). On the other hand, follow-up imaging with chest CT after discontinuation of adalimumab (Figures 3, 4) confirmed a reduction in the size of the lymphadenopathy clusters. The literature does in fact suggest an average interval of 22 months (ranging from three weeks to seven years) between the initiation of the anti-TNF agent and the development of a DISR (a rare side effect with an estimated incidence of one in 2,800). The anti-TNF agent most commonly implicated in this reaction is etanercept, followed by adalimumab and infliximab. The lungs are the most commonly affected organs, followed by the skin and eyes. Improvement of symptoms or complete resolution of the reaction is achieved with the discontinuation of the anti-TNF agent [2,4,6,8].

Adalimumab is an anti-TNF agent used in the treatment of autoimmune diseases such as psoriatic arthritis and sarcoidosis. However, paradoxically, adalimumab can cause a sarcoidosis-like reaction as a side effect. The reasons for this paradox remain uncertain, but it has been postulated that there may be an interaction between environmental factors, genetic characteristics, and immune responses [4,6,9]. In addition to anti-TNF agents (which include adalimumab), three other drug classes are considered capable of a DISR: immune checkpoint inhibitors, highly active antiretroviral therapy, and interferon [5].

## Conclusions

The authors propose that the sarcoidosis-like reaction observed in the patient was a consequence of the anti-TNF agent adalimumab used to treat the patient’s psoriatic arthritis. This hypothesis is supported by the temporal relationship between the initiation of the drug and the onset of symptoms, the histopathological analysis of the lymph node tissue, and the improvement in imaging findings following discontinuation of the medication. Although DISRs are uncommon and their pathophysiology remains unclear, this case aims to alert clinicians to consider this diagnosis in patients with similar clinical presentations who are being treated with anti-TNF agents or any other pharmacological class mentioned.
